# Exploring the Impact of Alcohol and Other Drug Use on HIV Care Among African American Older Adults Living with HIV/AIDS

**DOI:** 10.1093/geroni/igab046.3151

**Published:** 2021-12-17

**Authors:** Lesley Harris, Sydney Silverstein, Timothy Crawford, Jelani Kerr, Diana Ball

**Affiliations:** 1 University of Louisville, Louisville, Kentucky, United States; 2 Wright State University, Dayton, Ohio, United States

## Abstract

For people living with HIV, there are multiple barriers to engagement with care. This study qualitatively examines the role of use of alcohol and other drugs (AOD) on the health and management of Human Immunodeficiency Virus (HIV) disease among older African Americans (≥50 years). It draws on interviews conducted with twenty-seven older African Americans living with HIV in the Louisville, Kentucky area. Interviews were transcribed verbatim and then analyzed using constructivist grounded theory analytic techniques. Participants’ understandings of their AOD use fell on a continuum of problematic use to use for self-care. Regardless of where participants fell on this continuum, they faced a) environmental impacts of AOD use and b) current or historic discrimination from the health care system. The analysis focused on gaining a deeper understanding of the intersection of AOD use and engagement in the HIV care continuum. This revealed six major phases, which occurred at various stages of the continuum: (1) Linking AOD use as the cause of HIV diagnosis (2) Having AOD use facilitate denial of HIV, (3) Experiencing problematic use, (4) “Testing the Waters,” (5) Relying on AIDS Service Organizations (ASO) and medical providers and (6) Maintaining health and/or using AOD for self-care. We discuss the ways that stigma along the lines of race, gender, and age intersect with co-occurring conditions such as substance use disorders in complex and multifaceted ways. Recommendations include assessing a patients’ AOD use in relationship to the HIV care continuum to assess patients’ experiences and barriers within systems of care.

